# Supplementary material for: The Regulatory Cross-Talk between microRNAs and Novel Members of the B7 Family in Human Diseases: A Scoping Review
Source: Int J Mol Sci. 2021 Mar 6;22(5):2652. doi: 10.3390/ijms22052652 (PMC7962059; doi:10.3390/ijms22052652)
Supplement: Supplementary file 1 [file ijms-22-02652-s001.pdf]

PubMed search history.			
Search number	Query	Filters	Results
18	#16 AND #17		45
17	#14 OR #15		129,732
16	#1 OR #2 OR #3 OR #4 OR #5 OR #6 OR #7 OR #8 OR #9 OR #10 OR #11 OR #12 OR #13		2,820
15	"MicroRNAs"[Mesh]		83,142
14	((((((((((((((((((((((((((MicroRNA[Title]) OR (MicroRNA[Title/Abstract])) OR (MicroRNAs[Title])) OR (MicroRNAs[Title/Abstract])) OR ("Micro RNA"[Title])) OR ("Micro RNA"[Title/Abstract])) OR ("Micro RNAs"[Title])) OR ("Micro RNAs"[Title/Abstract])) OR (miRNA[Title])) OR (miRNA[Title/Abstract])) OR (miRNAs[Title])) OR (miRNAs[Title/Abstract])) OR (pri-miRNA[Title])) OR (pri-miRNA[Title/Abstract])) OR (stRNA[Title])) OR (stRNA[Title/Abstract])) OR ("Small Temporal RNA"[Title])) OR ("Small Temporal RNA"[Title/Abstract])) OR (pre-miRNA[Title])) OR (pre-miRNA[Title/Abstract])) OR (miR[Title])) OR (miR[Title/Abstract])) OR (miRs[Title])) OR (miRs[Title/Abstract])) OR (MIRN[Title])) OR (MIRN[Title/Abstract])) OR (MIRNS[Title])) OR (MIRNS[Title/Abstract])) OR (hsa-miR[Title])) OR (hsa-miR[Title/Abstract])		125,487
13	(((((((ILDR2[Title]) OR (ILDR2[Title/Abstract])) OR ("immunoglobulin like domain containing receptor 2"[Title])) OR ("immunoglobulin like domain containing receptor 2"[Title/Abstract])) OR (C1orf32[Title])) OR (C1orf32[Title/Abstract])) OR (dJ782G3.1[Title])) OR (dJ782G3.1[Title/Abstract])		16
12	"HHLA2 protein, human" [Supplementary Concept]		17
11	(((((((B7H7[Title]) OR (B7H7[Title/Abstract])) OR (B7-H7[Title])) OR (B7-H7[Title/Abstract])) OR (HHLA2[Title])) OR (HHLA2[Title/Abstract])) OR ("HERV-H LTR-associating 2"[Title])) OR ("HERV-H LTR-associating 2"[Title/Abstract])		49
10	"NCR3LG1 protein, human" [Supplementary Concept] OR "B7-H6 antigen, human" [Supplementary Concept]		44
9	(((((((((((B7H6[Title]) OR (B7H6[Title/Abstract])) OR (B7-H6[Title])) OR (B7-H6[Title/Abstract])) OR ("B7 homolog 6"[Title])) OR ("B7 homolog 6"[Title/Abstract])) OR (NCR3LG1[Title])) OR (NCR3LG1[Title/Abstract])) OR (DKFZp686O24166[Title])) OR (DKFZp686O24166[Title/Abstract])) OR ("natural killer cell cytotoxicity receptor 3 ligand 1"[Title])) OR ("natural killer cell cytotoxicity receptor 3 ligand 1"[Title/Abstract])		106
8	"BTNL2 protein, human" [Supplementary Concept]		78
7	(((((((((((((BTNL2[Title]) OR (BTNL2[Title/Abstract])) OR (BTL-II[Title])) OR (BTL-II[Title/Abstract])) OR ("butyrophilin-like MHC class II"[Title])) OR ("butyrophilin-like MHC class II"[Title/Abstract])) OR (BTLII[Title])) OR (BTLII[Title/Abstract])) OR (BTN7[Title])) OR (BTN7[Title/Abstract])) OR (HSBLMHC1[Title])) OR (HSBLMHC1[Title/Abstract])) OR ("butyrophilin like 2"[Title])) OR ("butyrophilin like 2"[Title/Abstract])		131
6	"VSIR protein, human" [Supplementary Concept]		46
5	(((((((((((((((((((((B7H5[Title]) OR (B7H5[Title/Abstract])) OR (B7-H5[Title])) OR (B7-H5[Title/Abstract])) OR ("V-domain Ig suppressor of T cell activation"[Title])) OR ("V-domain Ig suppressor of T cell activation"[Title/Abstract])) OR (VISTA[Title])) OR (VISTA[Title/Abstract])) OR		1,603

	(Dies1[Title])) OR (Dies1[Title/Abstract])) OR (C10orf54[Title])) OR (C10orf54[Title/Abstract])) OR (VSIR[Title])) OR (VSIR[Title/Abstract])) OR (DD1alpha[Title])) OR (DD1alpha[Title/Abstract])) OR (GI24[Title])) OR (GI24[Title/Abstract])) OR (PD-1H[Title])) OR (PD-1H[Title/Abstract])) OR (PP2135[Title])) OR (PP2135[Title/Abstract])) OR (SISP1[Title])) OR (SISP1[Title/Abstract])		
4	"V-Set Domain-Containing T-Cell Activation Inhibitor 1"[Mesh] OR "VTCN1 protein, human" [Supplementary Concept]		251
3	((((((((((((((B7H4[Title]) OR (B7H4[Title/Abstract])) OR (B7-H4[Title])) OR (B7-H4[Title/Abstract])) OR (B7x[Title])) OR (B7x[Title/Abstract])) OR (B7S1[Title])) OR (B7S1[Title/Abstract])) OR ("V-set domain containing T cell activation inhibitor 1"[Title])) OR ("V-set domain containing T cell activation inhibitor 1"[Title/Abstract])) OR (VTCN1[Title])) OR (VTCN1[Title/Abstract])) OR (VCTN1[Title])) OR (VCTN1[Title/Abstract])) OR (PRO1291[Title])) OR (PRO1291[Title/Abstract])		439
2	"CD276 protein, human" [Supplementary Concept]		256
1	(((((((((B7H3[Title]) OR (B7H3[Title/Abstract])) OR (B7-H3[Title])) OR (B7-H3[Title/Abstract])) OR ("B7 homolg 3"[Title])) OR ("B7 homolg 3"[Title/Abstract])) OR (CD276[Title])) OR (CD276[Title/Abstract])) OR (4Ig-B7-H3[Title])) OR (4Ig-B7-H3[Title/Abstract])		630

Embase search history.		
No.	Query	Results
#18	#16 AND #17	96
#17	#14 OR #15	181266
#16	#1 OR #2 OR #3 OR #4 OR #5 OR #6 OR #7 OR #8 OR #9 OR #10 OR #11 OR #12 OR #13	5149
#15	'microrna'/exp	165210
#14	microrna:ti,ab OR micrornas:ti,ab OR 'micro rna':ti,ab OR 'micro rnas':ti,ab OR mirna:ti,ab OR mirnas:ti,ab OR 'pri mirna':ti,ab OR strna:ti,ab OR 'small temporal rna':ti,ab OR 'pre mirna':ti,ab OR mir:ti,ab OR mirs:ti,ab OR mirn:ti,ab OR mirns:ti,ab OR 'hsa mir':ti,ab	162180
#13	ildr2:ti,ab OR 'immunoglobulin like domain containing receptor 2':ti,ab OR c1orf32:ti,ab OR dj782g3.1:ti,ab	28
#12	'hhla2 protein'/exp	13
#11	b7h7:ti,ab OR 'b7 h7':ti,ab OR hhla2:ti,ab OR 'herv-h ltr-associating 2':ti,ab	76
#10	'b7 h6 protein'/exp OR 'b7 h6 antigen'/exp	35
#9	b7h6:ti,ab OR 'b7 h6':ti,ab OR 'b7 homolog 6':ti,ab OR ncr3lg1:ti,ab OR dkfzp686o24166:ti,ab OR 'natural killer cell cytotoxicity receptor 3 ligand 1':ti,ab	154
#8	'btnl2 gene'/exp OR 'btnl2 protein'/exp OR 'butyrophilin like 2'/exp OR 'butyrophilin like 2 protein'/exp	90
#7	btnl2:ti,ab OR 'btl ii':ti,ab OR 'butyrophilin-like mhc class ii':ti,ab OR btlii:ti,ab OR btn7:ti,ab OR hsblmhc1:ti,ab OR 'butyrophilin like 2':ti,ab	201
#6	'v domain ig suppressor of t cell activation'/exp	15
#5	b7h5:ti,ab OR 'b7 h5':ti,ab OR 'v-domain ig suppressor of t cell activation':ti,ab OR vista:ti,ab OR dies1:ti,ab OR c10orf54:ti,ab OR vsir:ti,ab OR dd1alpha:ti,ab OR gi24:ti,ab OR 'pd 1h':ti,ab OR pp2135:ti,ab OR sisp1:ti,ab	3070
#4	'v set domain containing t cell activation inhibitor 1'/exp OR 'b7 h4 gene'/exp OR 'vtcn1 protein human'/exp	527
#3	b7h4:ti,ab OR 'b7 h4':ti,ab OR b7x:ti,ab OR b7s1:ti,ab OR 'v-set domain containing t cell activation inhibitor 1':ti,ab OR vtcn1:ti,ab OR vctn1:ti,ab OR pro1291:ti,ab	693
#2	'b7h3 protein'/exp OR 'b7 h3 protein'/exp OR 'b7 h3 antigen'/exp OR 'b7 h3 gene'/exp OR 'b7 homolog 3 protein'/exp OR 'cd276 antigen'/exp OR 'cd276 protein human'/exp OR 'cd276 gene'/exp	354
#1	b7h3:ti,ab OR 'b7 h3':ti,ab OR 'b7 homolg 3':ti,ab OR cd276:ti,ab OR '4ig b7 h3':ti,ab	1061
